# Cyberbullying and Non-Suicidal Self-Injury (NSSI) in Adolescence: Exploring Moderators and Mediators through a Systematic Review

**DOI:** 10.3390/children11040410

**Published:** 2024-03-29

**Authors:** Elena Predescu, Iulia Calugar, Roxana Sipos

**Affiliations:** 1 Department of Neuroscience, Psychiatry and Pediatric Psychiatry, “IuliuHatieganu” University of Medicine and Pharmacy, Republicii Street No. 57, 400489 Cluj-Napoca, Romania; predescu.elena@umfcluj.ro; 2 Clinic of Pediatric Psychiatry and Addiction, Clinical Emergency Hospital for Children, 400489 Cluj-Napoca, Romania

**Keywords:** cyberbullying, non-suicidal self-injury, adolescents, internalizing symptoms, protective factors

## Abstract

(1) Objective: This systematic review explores the intricate relationship between cyberbullying and non-suicidal self-injury (NSSI) in adolescents, acknowledging the dynamic nature of these phenomena in the evolving landscape of technology and social norms. (2) Methods: PubMed/MEDLINE, Web of Science, and EMBASE were searched, and 14 studies were selected based on the eligibility criteria, focusing on participants aged 10 to 19, cyberbullying roles, and NSSI as the predictor and outcome variables, respectively. (3) Results: Internalizing symptoms, specifically depression and anxiety, emerged as the most prominent mediators. However, factors such as externalizing symptoms, stress, and negative emotional responses (emotion reactivity, negative emotions) were also identified to play a significant role in the relationship between cyberbullying and NSSI. On the other hand, protective factors against the negative impact of cyberbullying on NSSI risk, such as strong peer connections and school engagement, were identified. (4) Discussions: This review underscores the multidimensional nature of the cyberbullying–NSSI association, emphasizing the roles of potential risk factors such as internalizing and externalizing symptoms, stress, and negative emotional response. Internalizing symptoms played a central role as pathways between cyberbullying victimization and NSSI. Additionally, social factors, including peer connections and school engagement, were found to act as protective elements. (4) Conclusion: Continuous investigation is crucial in order to adapt interventions to the evolving technological and social landscape. The study advocates for targeted interventions that prioritize positive social connections to mitigate the impact of cyberbullying on adolescent well-being.

## 1. Introduction

In today’s digital world, technology plays a pivotal role in shaping our daily lives, including the way we communicate and socialize. However, alongside its numerous benefits, it introduces new avenues for negative or harmful conducts, such as cyberbullying.

In the last decade, cyberbullying has become a public health issue, especially among adolescents. It represents one of the less desirable effects of technological evolution with a negative impact on mental health and the quality of life. It is defined as “an aggressive, intentional act carried out by a group or individual using electronic forms of contact, repeatedly and over time against a victim who cannot easily defend himself or herself” [1]. Cyberbullying includes but is not limited to harassment of others through text message, email, or social media, posting or sharing harmful or negative content about others, or even creating fake social media profiles meant to impersonate other people. With the rapid emergence of different online platforms, social media has become the most common vehicle for cyberbullying and its perpetration [2]. There are common elements between traditional bullying and cyberbullying, such as repetitiveness, power imbalance between the aggressor and the victim, intentionality, and aggressiveness [1]. However, contrary to the classical forms of bullying, cyberbullying is characterized by anonymity, a lack of need for physical interaction in order to produce harm and, moreover, by a non-stop availability of access to victims through electronic platforms [3]. Due to the characteristics of the environments in which cyberbullying occurs, this phenomenon has the potential of reaching a greater potential audience, as opposed to the classical form of bullying [4], which magnifies its potential harmful effects on cybervictims. Moreover, it has been shown that cyberbullies feel less remorse due to the fact that they cannot see their victims’ reactions [5], which contributes to significant perpetration of cyberbullying and further victimization through cyberbullying.

Thus, cyberbullying can be more harmful than other classical forms of bullying, as victims can be reached in their own homes and exposed to a wider audience with little or no adult supervision or authority entity present. Moreover, due to the aggressors’ anonymity and lack of fear of being caught [3], as well as a significant state of permanence of words or images disseminated in the online space [6], cyberbullying represents a public health problem that requires more resources in terms of prevention and intervention.

Research has identified potential predictors of cyberbullying victimization. A meta-analysis of predictors for both perpetration and victimization in cyberbullying has shown that previous experiences of victimization offline and psychological risk factors play significant roles in cyberbullying victimization [7]. It has been widely indicated that there is significant overlap between victims of traditional bullying and cybervictims [8], suggesting a carryover of traditional schoolyard bullying from the real world to the online world.

Common predictors for both cyberbullying and cyberbullying victimization are increased Internet use, poor family environment [9], low school commitment, and higher levels of impulsivity, narcissism, hostility, or/and phobic anxiety [2,7].

While boys are more likely to be involved in direct physical confrontation, bullying in the digital space is equally likely to be employed by girls as it is by boys [8]. Gender is one of the unique predictors that distinguishes between cybervictims and cyberbullies, with girls presenting a higher risk of cyberbullying victimization [7]. At an individual level, negative beliefs about the self, such as low self-esteem or self-concept [10], low agreeableness [11], high outgoingness and openness to risky behaviors [12], and being a member of the LBGTQ community [13] were related to being a cybervictim, while aggressors presented a lack of moral values, empathy, or remorse [5,7]. Social media behaviors such as posting indiscreet or negative content and a higher number of social media “friends” were also linked to victimization through cyberbullying [14].

Increased levels of psychological and physical distress have been reported among victims of cyberbullying. While most research has been focused on the internalizing aspects of the mental health issues generated by cyberbullying, such as anxiety, negative affect, or loneliness [15,16], a higher risk of non-suicidal self-injurious acts in cybervictims has been identified across multiple studies over the last decade [17,18].

An important concern in self-injurious behavior research revolves around the terminology used, with expressions like “non-suicidal self-injury” (NSSI) and “self-harm” prevailing. NSSI is characterized as the “deliberate, self-inflicted destruction of body tissues, with no intention of suicide and no purpose of being sanctioned by society” [19]. Self-harm represents an encompassing term referring to “an intentional act of self-poisoning or self-injury, irrespective of the motivation or apparent purpose of the act” [20]. Thus, NSSI refers to a type of self-harm which involves no suicidal intent, while self-harm encompasses all types of non-fatal self-injurious acts, with or without suicidal intent [21,22]. Victimization has been identified as a contributing factor to NSSI in adolescents [23,24]. The conceptual framework of the interpersonal model of NSSI suggests that individuals facing adverse interpersonal events may adopt NSSI as an adaptive mechanism to cope with stress, tension, or to seek relief from distressing experiences [19]. Shedding light on the pathways between stressful life events and NSSI, Zhou et al. (2024) indicates multiple mechanisms. One indirect pathway involves internalizing symptoms as a mediating factor of this relationship, while a second, more complex pathway involves co-mediation by both dysfunctional emotion regulation and internalizing symptoms. Moreover, a direct association between recent stressful life events and NSSI was also observed [25]. With the escalating number of Internet users, cyberbullying victimization is increasingly prevalent, particularly among adolescents [26]. A recent study investigating the relationship between peer victimization and NSSI in 14.666 high school students indicated that peer victimization directly predicts NSSI. Moreover, it has shown that social anxiety and mobile phone use presented mediating effects between peer victimization and NSSI [27]. Data related directly to cyberbullying shows that cybervictims exhibit a greater risk of self-harm than non-victims. Similarly, but to a lesser extent, cyberbullying perpetrators are at a greater risk of self-harm compared to non-perpetrators [28].

Building on the concept of risk and protective factors, Baker et al. (2023) investigated how adolescents’ perceptions of socioecological connectedness influence the likelihood of NSSI. Their finding revealed that stronger family connectedness both directly and indirectly reduced the risk of NSSI through bullying victimization and depressive symptoms, while school connectedness showed an indirect protective effect [29].

A comprehensive literature review investigating the global prevalence of cyberbullying revealed an ascending trend in the prevalence rate over a 5-year period of study from 2015 to 2019. The average cyberbullying perpetration rate indicated was 25.03%, while the average victimization rate was 33.08% [30]. It was observed that these rates showed higher values when compared to previous similar research, with percentages of cyberbullying involvement ranging from 15% to 25% [31,32].

Extensive evidence supports the association between cyberbullying and an increased likelihood of individuals, both victims and perpetrators, engaging in self-injurious behaviors like cutting or burning oneself [28]. The prevalence of self-injurious behaviors, regardless of the cause, has evolved into a public health concern, exerting lasting negative impacts on the lives of those involved, both in the subsequent years and extending into adulthood [33]. The lifetime prevalence of self-injurious behaviors both with and without suicidal intent has been reported at 20% in a meta-analytical study investigating the global prevalence of self-harming behaviors in adolescents [34]. Farkas et al. (2023) revealed in their recent systematic review and meta-analysis an overall prevalence of NSSI of 16% in studies published between 2015 and 2020. Noteworthy gender differences in prevalence rates were also reported, with a prevalence rate of approximately 21% for females and 16.5% for males [35]. A 90% prevalence of internet use has been reported among adolescents that employ self-injurious acts [36]. Other than being a venue for bullying perpetration, the digital space can potentially normalize self-harming behaviors and impact the repetitiveness of these behaviors [28]. These findings emphasize the pressing need for comprehensive research to explore the intricate dynamics between cyberbullying and NSSI among adolescents, considering its far-reaching impact on adolescent well-being.

### The Present Study

The data presented above suggest a significant association between cyberbullying involvement and NSSI engagement. This systematic review aims to investigate moderating and mediating variables between cyberbullying and NSSI, taking into consideration the status of involvement (victim/aggressor). Previous research conducted by Moore et al. [37] investigated the relationship between the types of bullying involvement and self-harmful thoughts and behaviors in young people, revealing significant data heterogeneity and a noteworthy lack of terminological consistency within the literature. To our current knowledge, no systematic review to date has exclusively addressed the non-suicidal dimension of self-harming behaviors in adolescents involved in cyberbullying. Consequently, our objective is to systematically identify the mediator and moderator variables that influence the relationship between cyberbullying and NSSI occurrence. The current review investigates the following question: what are the moderating and mediating variables associated with NSSI in adolescents involved in cyberbullying? This analysis aims to provide a comprehensive understanding of the nuanced interplay between cyberbullying and NSSI among adolescents, contributing valuable insights to the existing body of knowledge.

## 2. Materials and Methods

This review follows the Preferred Reporting Items for Systematic Reviews and Meta-Analyses (PRISMA) guidelines [38,39,40] (Supplementary Figure S1).

### 2.1. Information Sources

Comprehensive searches for the relevant literature were conducted across multiple academic databases: PubMed/MEDLINE, Web of Science, and EMBASE. The search strings specific to every database employed controlled cyber-related vocabulary terms in order to ensure a robust and exhaustive retrieval of relevant studies (see Table 1), minimizing the risk of publication bias and guaranteeing the comprehensiveness of this systematic review. The strings used for each academic database can be found in Supplementary Table S1.

### 2.2. Eligibility Criteria

A structured approach based on PICOS principles [41] was employed to determine the study inclusion criteria.

In accordance with the World Health Organization’s definition of adolescence [42], participants aged 10 to 19 were recruited for this study. The sample populations encompassed individuals identified as victims or aggressors in the context of cyberbullying, with participants identified as bystanders excluded. No eligibility criteria were established based on other participant characteristics. Studies which examined cyberbullying independently were included. Studies which investigated cyberbullying and other forms of bullying were included only if they ensured a clear differentiation between the types of aggression approached. It was required that studies investigate at least one of the following cyberbullying roles as a predictor variable: victim, aggressor, or aggressor–victim. Additionally, studies were required to examine NSSI as an outcome variable. Taking into consideration the significant overlap in definitions, studies investigating self-harm behaviors explicitly measured and reported as lacking suicidal intent were included. Our analysis relied on full-text, peer-reviewed articles with original research data, published in English and designed as observational studies (cross-sectional, case–control, or longitudinal). This excluded book chapters, case studies, non-peer-reviewed materials, theses, dissertations, systematic reviews, meta-analyses, research protocols, and opinion pieces.

### 2.3. Study Selection

The initial search of bibliographic databases yielded 323 records. Upon the removal of 116 duplicates, the titles and abstracts of 207 records were screened. The first and second author conducted a screening of the titles and abstracts in the Zotero reference management software v4.0. This initial screening aimed to identify studies that met the predefined inclusion criteria based on the title and abstract content. Forty-one of the full-text studies retrieved were evaluated for eligibility by the first and second authors. Discrepancies in eligibility assessment were resolved through structured discussions involving the third author until consensus was reached. Following a rigorous selection process based on the predetermined inclusion criteria, 27 of the 41 retrieved studies were excluded. A detailed overview for each excluded study, along with the specific criteria that were not met, is provided in Section 3.1, as well as Supplementary Figure S1: Quality assessment and inter-rater reliability.

In order to ensure quality control, two researchers independently assessed study quality using the Newcastle–Ottawa Scale (NOS). The NOS for non-randomized case–control and cohort studies [43] and the adapted version for cross-sectional studies developed by Herzog and colleagues were used to establish methodological quality [44]. Quality assessment adhered to a star-based system, with case–control and cohort studies categorized on a 0–9 scale and cross-sectional studies rated on a 0–10 scale. Discrepancies were resolved through consensus discussion. Studies scoring ≤5 on the NOS were deemed to have a high risk of bias, while those scoring ≥6 were categorized as having a low risk of bias. Inter-rater reliability, measured as the percentage of agreement, was calculated as the number of agreements divided by the total number of scores.

### 2.4. Data Extraction and Analysis

To address the research question, data were extracted from the included studies for the following key categories: author and year of publication, type of design, population (sample size, mean age, gender distribution, household income, area type), bullying participation role, measure of cyberbullying, outcome variables, measures of outcomes, moderator and mediator variables, measure instruments of moderators/mediators, and timespan of measurement.

Descriptive statistics were computed for key sample characteristics. These characteristics included sample size, mean age, gender distribution, categorized household income levels, and area type (urban/rural). Data on these characteristics were extracted from the included studies and compiled into a database using IBM SPSS Statistics software V.17. In addition, the studies included in this review employed a variety of methodologies (e.g., surveys, interviews, validated psychometric instruments) in order to measure NSSI as well as cyberbullying. This fact, coupled with the relatively limited number of articles identified through our search strategy, rendered a meta-analysis infeasible.

## 3. Results

### 3.1. Overview and Characteristics of Included Studies

Full-text assessment resulted in the exclusion of 27 studies that did not comply with the predetermined inclusion criteria, as outlined in the methodology section. Seventeen studies were excluded from analysis due to the outcome variable not aligning with the established criteria (e.g., unspecified lack of suicidal intent) [45,46,47,48,49,50,51,52,53,54,55,56,57,58,59,60,61]. Four additional studies [62,63,64,65] were excluded because cyberbullying was not measured independently from other types of bullying or mistreatment. Other four studies were excluded because the predictor and outcome variables did not meet the inclusion criteria; one study [66] did not investigate cyberbullying as a predictor variable, while the other three studies [67,68,69] did not assess NSSI as an outcome variable. The exclusion of [18,70] was due to study participants exceeding the upper age limit (19 years old). A final selection of 20 articles was considered for further analysis. Of the fourteen studies included, twelve were cross-sectional, while two were longitudinal (see Table 2).

### 3.2. Populations

The studies included participants ranging in age from 10 to 19 years old (M = 14.30, SD = 1.27). Sample sizes varied from 64 to 2647.

Half of the studies (n = 7) included participants from China, whilst others were carried out on American (n–2), Australian (n = 2), Iranian, Indonesian, and Hungarian populations. Two studies included in this review used the same participant sample in their respective analyses [26,84]. Most studies (n = 12) reported gender characteristics. Using the available data, it was calculated that females comprised 52.29% of the population (Mean = 584.91, SD = 120.37), whilst males comprised 47.70% (Mean = 581.33, SD = 131.88). Regarding sociodemographic aspects, only half of the studies reported area characteristics, while only four reported household income levels. Thus, using the data available, it was revealed that 67.5% of the participants resided in urban areas (Mean = 917.85, SD = 191.62), while 32.49% resided in suburban or rural areas (Mean = 546.14, SD = 204.87). Low-income households accounted for 20.27% of the total (Mean = 330.90, SD = 119.17), while medium and high-income households accounted for 79.63% (Mean = 1164.11, SD = 276.56) One study focused on a specific population—students with intellectual disabilities [93]. Only one study utilized a clinical sample [79], while the remaining studies recruited participants from educational settings.

### 3.3. Predictor Variables

Eight studies solely assessed cyberbullying, whereas six assessed both traditional bullying and cyberbullying. All joint studies conducted separate analyses for each of the two types of bullying.

Data on cyberbullying experiences exclusively relied on self-reported measures across studies, adopting diverse operational definitions and psychometric instruments. These ranged from basic questionnaires providing definitions or examples of cyberbullying acts [79,86] to validated psychometric instruments [71,84,98], or even adapted items from established tools [76,90]. Psychometric properties were reported in eight of the fourteen studies included.

### 3.4. Outcome Variables

As shown in Table 2, outcome variables were measured differently across the included studies. Ten studies utilized the term “NSSI” specifically, whereas others operationalized the construct by emphasizing the lack of suicidal intent in self-harm behaviors within their methodologies. Eight studies measured single outcomes (NSSI), whereas six measured additional outcomes alongside NSSI (e.g., suicidal ideation, suicide attempts, substance use). Notably, all studies conducted separate analyses for NSSI, regardless of the number of variables.

Outcome variables were collected exclusively through self-report data across all studies. Self-report measures included single-item questions, standardized questionnaires, validated psychometric instruments, or adapted items from a validated tool. Measure reliability characteristics were mentioned for eight of the included studies.

### 3.5. Quality Assessment

Quality assessment scores varied significantly, with 10 studies fulfilling the established criteria for low risk of bias. Detailed explanations of potential bias sources across studies with lower quality assessment scores are provided in Supplementary Table S2.

### 3.6. The Association between the Role of Cybervictim and NSSI

Regarding individual factors, several studies investigated internalizing symptoms as mediators in the relationship between cyberbullying victimization and NSSI [73,81,84,98]. Internalizing symptom assessments included various indicators such as overall internalizing symptom scores [73], measurements of depression [84], anxiety [98], or self-esteem [81] (see Table 3).

It has been indicated that online victimization indirectly contributes to both past NSSI (indirect effect = 0.044; SE = 0.01; *p* < 0.01) and current NSSI (indirect effect = 0.058; SE = 0.04; *p* < 0.001) via internalizing problems in a sample of high-school students, with a slightly larger effect size for current NSSI. Regarding internalization, self-esteem emerged as a partial mediator in the relationship between cybervictimization and NSSI; specifically, cybervictimization was associated with lower self-esteem (β = −0.21, *p* < 0.05), and lower self-esteem, in turn, was associated with higher NSSI (β = 0.32, *p* < 0.05) in high-school-aged participants [81]. The results also indicated that cybervictimization leads to NSSI indirectly through increased depressive symptoms (indirect effect = 0.09, SE = 0.02, 95% CI [0.06, 0.14]) [84], while anxiety mediated this association in adolescents with low self-control [98] in samples comprising middle-school students. Similarly, externalizing symptoms were also shown to mediate the relationship between cyberbullying victimization and NSSI (β = 0.011; SE = 0.01; *p* < 0.05). Moreover, additional cross-sectional studies have identified various mediators involved in the relationship between cyberbullying victimization and NSSI such as school engagement [26] but also emotional reactivity (β = 0.05, 95% CI = [0.031, 0.061]) [66], negative emotions (β = 0.04, SE = 0.01, 95% CI [0.02, 0.06]) [105] and stress [90], thus highlighting the role of negative emotions in adolescent populations.

Social factors. In terms of social support, strong peer connections can buffer against the negative impact of cyberbullying on self-esteem and NSSI risk, influencing the self-esteem pathway (β = 0.013, 95% CI [0.004, 0.025]) in high-school-aged samples [81]. Moreover, feelings of school connectedness reduced the influence of depression on mediating the link between cybervictimization and NSSI (indirect effect = 0.12, SE = 0.03, 95% CI [0.07, 0.18]) [84], while high-quality friendships buffered the effect of negative emotions on the relationship between cybervictimization and NSSI (β = −0.04, 95% CI [−0.09, −0.002]) in middle-school samples [87].

### 3.7. The Association between the Role of Cyberbully and NSSI

Only two studies [73,90] investigated the relationship between the role of cyberbully and NSSI. Externalizing problems mediated the association between the role of online bully and NSSI (β = 0.022; SE = 0.01; *p* < 0.01), as well as between the role of online bully–victim and NSSI (β = 0.028; SE = 0.01; *p* < 0.01) [73]. Stress was also highlighted as a partial mediator [90].

## 4. Discussion

The significance of this systematic review is underscored by the dynamic and evolving nature of both cyberbullying and non-suicidal self-injury among adolescents. With the earliest study included in this review dating to 2019, the rapid pace of technological advancement and evolving social norms require continuous investigation on this critical topic. While there is considerable understanding of the impact of cyberbullying victimization on adolescent NSSI, it is recognized that not all adolescents are uniformly affected by such victimization [113]. Consequently, potential moderators that either mitigate or intensify the risk associated with cyberbullying victimization on adolescent NSSI must exist. By comprehensively synthesizing the current research, this review aims to provide an up-to-date understanding of the complex interactions between cyberbullying and NSSI in this vulnerable population via identifying mediating and moderating variables between cyberbullying participation roles and NSSI in adolescents.

The relationship between cybervictimization and NSSI emerged as the most prominent theme in this systematic review, as it is the focus of most records identified. Notably, our analysis identified a multitude of mediators and moderators that extend the findings of previous studies. Research has established a notable link between being subjected to bullying and increased stress levels among adolescents. This heightened stress, in turn, appears to correlate with a higher likelihood of engaging in NSSI as a coping mechanism. The connection between bullying, stress, and NSSI suggests that adolescents may resort to self-injurious behaviors to manage or alleviate the emotional distress brought about by the bullying experience [23]. Furthermore, this association intertwines with the concept of internalizing problems, which encompass various emotional and psychological challenges faced by individuals. Adolescents who experience cyberbullying may internalize the negative effects of online harassment, leading to heightened stress levels and, subsequently, an increased propensity for engaging in NSSI [114]. At an individual level, internalizing symptoms played a central role as pathway between cyberbullying victimization and NSSI in adolescents. Overall, internalizing problems significantly mediated the relationship between online victimization and both past and current NSSI, suggesting that experiencing cyberbullying can contribute to NSSI risk through increased internal distress [73]. Within the spectrum of internalizing symptoms, depression emerged as playing a role in the interplay of cyberbullying and NSSI, suggesting that cyberbullying victimization can trigger depressive symptoms, which can then increase the risk of NSSI [84]. This supports existing evidence on the relationship between one’s involvement in cyberbullying and affective symptoms [15,16,115]. An interesting finding regarding the internalizing symptomatology is that anxiety symptoms only mediated the relationship between cyberbullying and NSSI in adolescents with low self-control in a longitudinal study [98]. Empirical evidence suggests that cybervictimization heightens the risk of anxiety symptoms in adolescents [116,117]. Also, prior research demonstrated a positive link between anxiety symptoms and NSSI [118,119,120]. In addition, few studies supported the role of self-control as a moderator in the proposed mediation model, aligning with general strain theory (GST) [121]. Higher self-control, according to GST, enables adolescents to regulate impulsive reactions and negative emotions effectively in response to stress, a notion corroborated by existing evidence demonstrating the interaction between self-control and environmental risk in predicting adolescent internalizing problems [122]. However, the scarcity of studies on this specific mediating effect requires cautious interpretation and highlights the need for further research on the topic. Focusing on another specific internalizing symptoms, low self-esteem acted as a mediator, indicating that cyberbullying can lead to decreased self-esteem in victims, which in turn increases their vulnerability to engaging in NSSI behaviors [81]. Available data investigating the association between self-esteem and cyberbullying victimization indicate a bidirectional relationship. Cyberbullying victimization has been linked to decreased self-esteem, while high self-esteem has been indicated to be a protective factor against victimization [123,124]. Apart from internalizing symptoms, it was found that externalizing symptoms are also involved in the studied association. Although specific externalizing symptoms are not described, previous studies have indicated that both perpetrators and victims of cyberbullying engage in substance use or violence-related behaviors [125,126]. Cyberbullying victims may struggle with managing the intense emotions of anger, frustration, and helplessness brought on by victimization, which can in turn lead to externalizing these emotions through disruptive or aggressive behaviors [10]. Moreover, they may engage in externalizing behaviors as a means of retaliation, to regain a sense of control [127], or due to the normalizing effects of witnessing online aggression, which can contribute to the adoption of similar tactics and desensitization to their harmful impact [128]. Furthermore, the key role of negative emotional response was suggested in three of the studies identified [66,87,90]. Cybervictimization correlates with negative emotions in adolescents [129]. Emotional consequences include decreased self-esteem and increased anger, embarrassment, and grief [130]. Victims of cyberbullying exhibit higher levels of hostility, depressive symptoms, and persecution-related anxiety compared to non-victimized peers [114]. Klonsky (2007) notes NSSI is often seen as a negative emotional coping strategy [131]. In addition, Hughes et al. (2019) found that negative emotions, including anxiety, strongly predict adolescents’ NSSI [132]. Behavioral experiments suggest NSSI is triggered by guilt and shame, which it subsequently alleviates [59]. These findings were supported by a recent systematic review of risk and protective factors for self-harm behaviors among the adolescents involved in cyberbullying which indicated that higher self-control and emotional intelligence represent protective factors against bullying victimization. Moreover, it emphasized the role of perceived stress as a risk factor [133], which emerged as a moderator for both the roles of cybervictim and cyberbully.

Overall, these findings emphasize the multidimensional nature of the cyberbullying–NSSI association, with both internalizing and externalizing factors playing crucial roles as mediating and moderating factors in the association between NSSI and cyberbullying victimization and aggression.

From a social perspective, our findings highlight the importance of social support as a key component. It has been indicated that in the case of adolescents who exhibit high levels of attention-seeking behaviors, being engaged in school may act as a buffer against the negative consequences of cyberbullying, potentially reducing their risk of engaging in NSSI [26]. Peer connection-related findings have indicated positive peer relationships can support self-worth and reduce the impact of cybervictimization [81]. Furthermore, it has been indicated that feeling integrated within the school environment can buffer against the depressive symptoms triggered by cyberbullying experiences [84]. High-quality friendships were found to directly mitigate the effect of negative emotions on the cybervictimization–NSSI association, further highlighting the important role of positive social connections [87]. A recent systematic review investigating cyberbullying prevention strategies underscores the critical role of schools in this initiative. Their findings reveal that the majority of existing interventions emphasize educational approaches, primarily targeting schools and families. These strategies often involve enhancing teacher and parent training, fostering a positive school climate, and integrating social–emotional learning programs [134]. Furthermore, school-based interventions were proven effective in reducing cyberbullying victimization and perpetration in another meta-analytical review assessing cyberbullying intervention and prevention programs [135]. Overall, these findings offer robust evidence for prioritizing interventions aimed at developing positive and supportive social connections that could counteract the impact of cyberbullying victimization on adolescent well-being.

## 5. Limitations

Although this review strengthens our understanding of the relationship between cyberbullying and NSSI by focusing on psychological symptoms and social factors, it is important to acknowledge some limitations. Considering that this is a relatively new field of research, there is a restricted number of studies meeting our inclusion criteria. This potentially limits the generalizability of our findings and the ability to comprehensively assess the existing evidence base. Moreover, existing research investigating the association between cyberbullying and self-harm behaviors encompasses a broader spectrum of self-injurious behaviors, including both NSSI and suicidal behaviors. However, our review focused solely on NSSI, potentially excluding relevant studies that do not explicitly distinguish between suicidal and non-suicidal self-harm behaviors.

Most studies included used a cross-sectional design, limiting causal inferences and understanding of temporality. While these studies provide valuable insights into the association between cyberbullying and NSSI, they cannot definitively demonstrate whether cyberbullying causes NSSI or vice versa. Further research employing longitudinal designs is needed to explore the developmental pathways and potential bidirectional influences involved. Moreover, a cross-sectional design lacks an adequate control for confounding variables, potentially overseeing variables that might influence both cyberbullying and NSSI. Thus, the exploration of this topic in cohort studies is required to address this limitation.

Several limitations related to operationalizing definitions and measurement of key variables were identified. Despite some shared methodological approaches, the studies employed different conceptualizations and timeframes over which variables were measured as well as a diverse range of instruments to measure both cyberbullying and NSSI. This heterogeneity in measurement strategies poses a significant challenge, limiting the comparability of results. Additionally, not all studies reported the reliability and validity properties of their chosen instruments, raising concerns about the accuracy of their findings.

Such methodological inconsistencies pose challenges for data comparison, interpretation, and generalizability of findings across the field. Further research establishing a clearer distinction between NSSI and other self-harm behaviors as well as standardized measurements of cyberbullying are required. This would contribute to a more comprehensive understanding of the relationship between cyberbullying and NSSI.

## 6. Conclusions

This systematic review offers insights into the complexities of the relationship between cyberbullying and NSSI in adolescents, highlighting diverse factors that mitigate this association. While this review encountered significant heterogeneity in the literature, as well as a restricted body of literature, it revealed that factors such as internalizing symptoms and positive social connections play an important role in mediating the relationship between cyberbullying and NSSI. Although internalizing symptoms (depression, anxiety, decreased self-esteem) were the most prominent mitigating factors identified, we acknowledge the potential influence of other factors, such as externalizing symptoms, stress, and negative emotional responses (emotion reactivity, negative emotions). Thus, adolescents experiencing these symptoms may be more vulnerable to the negative psychological consequences of cyberbullying, potentially leading to increased NSSI behaviors. On the other hand, this review highlights the protective role of positive social connections (increased levels of peer attachment, school connectedness and engagement). Strong and supportive relationships with friends, family, or mentors can buffer the impact of cyberbullying and offer adolescents a sense of belonging and emotional security, ultimately reducing the risk of NSSI.

Furthermore, this review acknowledges the unpredictable nature of cyberbullying compared to traditional forms. Victims are often more exposed, easier to target, and lack the real-time supervision present in physical settings. This underscores the urgent need for effective prevention strategies tailored to the specific risks of cyberbullying. 

In conclusion, future research could benefit from exploring this relationship using longitudinal designs while ulteriorly investigating intervention development and effectiveness. Rigorous studies investigating the effectiveness of interventions specifically tailored to adolescents are required. Existing research demonstrates the effectiveness of school-based programs in preventing and intervening against cyberbullying. However, a critical gap remains in the lack of standardization across these programs. Further research is needed to establish a more unified approach, ensuring the consistent implementation of evidence-based strategies across diverse school settings. In this aspect, our findings could be valuable in the development of prevention strategies towards fostering positive social connections and equipping young people with effective emotion regulation skills.

## Figures and Tables

**Table 1:** Key terms employed in database search.

Term Category 1: Cyberbullying	Term Category 2: NSSI	Term Category 3: Child x Adolescent
Cyber-bull* Cyber-victim* Cyberstalk* Cyber harass* Online bully* Online victim* Online stalk* Online harass* Internet bully* Internet stalk* Internet harass* Electronic bully* Electronic stalk* Electronic harass*	NSSI Non-suicidal self-injury Self-harm* Self-injur* Automutilat*	Child* Adolescen* Youth School aged Student*

**Table 2:** Characteristics of the studies included in this systematic review.

Author, Year, Country	Type	Population	Population Characteristics	Bullying Participation Role	Cyberbullying Measure	Outcome Variables	NSSI Measure	QA
Azami and Taremian 2020 [71], Iran	Cross-sectional	School sample: High-school students	N = 400; Mean age = 16.61;	Traditional and cyberbullying victimization	Self-report: E-Victimization scale (Lam and Li 2013) [72], Cronbach’s alpha 0.85	Self-harm */Suicide attempts/Substance use	Single-item question	6
Drubina et al. 2023 [73], Hungary	Cross-sectional	School sample: high-school students	N = 1011, Mean age = 16.81, SD = 1.41	Traditional and cyberbullying aggression and victimization	Self-report: Revised Olweus Bully/Victim Questionnaire (Olweus 1996) [74]	NSSI	Self-report: Inventory of Statements About Self-Injury (ISAS) (Washburn et al., 2012) [75],	5
M. I. Islam, Khanam, and Kabir 2020 [76], Australia	Cross-sectional	School Sample: high-school students	N = 2166, Mean age = 14.83, SD = 1.70	Traditional and cyberbullying victimization	Self-report: Survey adapted from Olweus Bully-Victim Questionnaire and Cyber Friendly Schools Project (Cross et al., 2016 [77]; Thomas et al., 2017 [78])	Suicidal ideation/Self-harm*	Single-item question	6
M. Islam et al. 2021 [67], Australia	Cross-sectional	Community sample: adolescents	N = 2125, Mean age N.A. (age range 14–17)	Traditional and cyberbullying victimization	Single-item question	Suicidality/Self-harm *	Single-item question	4
Lanzillo et al. 2023 [79], USA	Cross-sectional	Clinical sample: inpatient and outpatient psychiatric adolescents	N = 64; Mean age = 14.3; SD = 1.44;	Cyberbullying victimization	Self-report: 3-item questionnaire	NSSI/Suicide attempt/Suicide threat/Suicidal ideation	Self-report: The Self-Harm Behavior Questionnaire (SHBQ) (Gutierrez et al., 2001 [80]) *Cronbach’s alpha* 0.89–0.96	5
Lin et al. 2023 [81], China	Longitudinal	School sample: high-school students	N = 1368. Mean age = 15.05, SD = 0.85 at T1	Cybervictimization	Self-report: 18-item cyberbullying victimization scale (Erdur-Baker and Kavsut, 2007 [82]) *Cronbach’s alpha* = 0.89	NSSI	Self-report: selected items from Deliberate Self-Harm Inventory (Gratz 2001 [83]) *Cronbach’s alpha* = 0.97	6
Liu et al. 2023 [84], China	Cross-sectional	School sample: middle-school students	N = 1006, Mean age = 13.16, SD = 0.67	Cybervictimization	Self-report using the Cyberbullying Victimization Scale (Erdur-Baker and Kavsut, 2007 [82]) *Cronbach’s alpha* = 0.82	NSSI	Self-report: Non-Suicidal Self-Injury scale (NSSI) (Yu et al., 2013 [85]) *Cronbach’s alpha* = 0.71	8
Peng et al. 2019 [86], China	Cross-sectional	School sample: middle- and high-school students	N = 2647, Mean age = 13.6, SD = 1.1	Traditional and cyberbullying victimization	Self-report: questionnaire	Suicide attempts/Suicidal ideation and self-harm/Suicidal ideation/Self-harm only	Single-item question	7
Y. Wang, Chen, and Ni 2021 [87], China	Cross-sectional	School sample: middle- and high-school students	N = 1324; Mean age = 13.67; SD = 1.34	Cyberbullying victimization	Self-report: subscale of the Cyberbullying Scale (Kwan and Skoric 2013 [88]) *Cronbach’s alpha* = 0.89	NSSI	Self-report: The Adolescent Self-Harm Scale (Feng 2008 [89]) *Kuder-Richardson* 0.83	6
Wiguna et al. 2021 [90], Indonesia	Cross-sectional	School sample: middle- and high-school students	N = 464; Mean age = 14.61; SD = 1.65	Cyberbullying victimization and aggression	Self-report: adapted three-item questionnaire (Hinduja and Patchin 2019 [91]; 2010; Sourander et al., 2010 [92]; Wiguna et al., 2018 [60]) *CR* = 0.958	NSSI	Self-report: adapted 3-item questionnaire (Sourander et al., 2010 [92]; Wiguna et al., 2018 [60]) *CR* = 0.953	4
Wright and Wachs 2020 [93], USA	longitudinal	School sample: adolescents with intellectual or developmental disabilities	N = 121; Mean age = 14.10;	Traditional and cyberbullying victimization and bystanding	Self-report: 12-item questionnaire (Wright and Li 2013 [94]; Wright, Wachs, and Harper 2018 [95])	Health complaints/Suicidal ideation/NSSI	Self-report: The Self-Harm Inventory (Sansone, Wiederman, and Sansone 1998 [96])	8
Yu et al. 2020 [26], China	Cross-sectional	School sample: middle-school students	N = 1006; Mean age = 13.16, SD = 0.67	Cybervictimization	Self-report: the Cyberbullying Victimization Scale (Erdur-Baker and Kavsut 2007 [82]) *Cronbach’s alpha* = 0.82	NSSI	Self-report: Non-Suicidal Self-Injury Scale (You et al., 2013 [85]) *Cronbach’s alpha* = 0.71	8
Zhao et al. 2022 [66], China	Cross-sectional	School sample: elementary and middle-school students	N = 2523; Mean age = 13.22, SD = 1.60	Cybervictimization	Self-report: Cyberbullying Victimization subscale of the second revision of the Revised Cyberbullying Inventory (Topcu and Erdur-Baker 2018 [97]) *Cronbach’s alpha* = 0.92	NSSI	Self-report: Deliberate Self-Harm Inventory (Gratz 2001 [83]) *Cronbach’s alpha* = 0.91	8
Zhu et al. 2021 [98], China	Longitudinal	School sample: middle-school students	N = 1987; Mean age T1 = 12.32, SD = 0.53 Mean age T2 = 12.82, SD = 0.53, Mean age T3 = 13.33, SD = 0.53	Cybervictimization	Self-report: Cybervictimization subscale in the Electronic Bullying Questionnaire (EBQ) at T1 (Moore, Huebner, and Hills 2012 [99]; Tian, Yan, and Huebner 2018 [100]) *Cronbach’s alpha* = 0.7	NSSI	Self-report: 7-item questionnaire *Cronbach’s alpha* = 0.8	7

* Refers to non-suicidal self-harm behaviors.

**Table 3:** Moderators and mediators between cyberbullying role and NSSI.

Cyberbullying Role	Author, Year, Country	Outcome	Timespan *	Moderators	Mediators	Measures
CV and CA	Drubina et al., 2023 [73], Hungary	NSSI	N.A.		Internalizing problems were a mediator between the role of online victim and past NSSI (β = 0.044; SE = 0.01; *p* < 0.01), online bully–victim and past NSSI (β = 0.032; SE = 0.01; *p* < 0.01), and online victim and current NSSI (β = 0.058; SE = 0.04; *p* < 0.001). Externalizing problems were a mediator between the role of online bully and current NSSI (β = 0.022; SE = 0.01; *p* < 0.01), online victim and current NSSI (β = 0.011; SE = 0.01; *p* < 0.05), and online bully–victim and current NSSI (β = 0.028; SE = 0.01; *p* < 0.01).	Self-report: The Strength and Difficulties Questionnaire (SDQ) [101]
CV	Lin et al., 2023 [81], China	NSSI	12 months	High peer attachment moderated the indirect effect of self-esteem on the relationship between cybervictimization and NSSI (β = 0.013, SE = 0.005, 95% CI = 0.004 to 0.025)	Self-esteem was a mediator between cybervictimization and NSSI (indirect effect = 0.007, SE = 0.002, 95% CI = 0.003 to 0.011)	Self-report: Self-Esteem Scale (SES) [102] and The Inventory of Peer Attachment Scale [103]
CV	Liu et al., 2023 [84], China	NSSI	Past 6 months	School connectedness moderated the indirect effect of depression on the association between cybervictimization and NSSI (indirect effect = 0.12, SE = 0.03, 95% CI [0.07, 0.18])	Depression significantly mediated the relationship between cybervictimization and adolescent NSSI (indirect effect = 0.09, SE = 0.02, 95% CI [0.06, 0.14])	Self-report: Depression was evaluated using The Center for Epidemiologic Studies Depression Scale [104] and the Emotional Engagement Sub-scale of the School Engagement Scale [105]
CV	Wang, Chen and Ni, 2021 [87], China	NSSI	N.A.	Friendship quality moderated the indirect association between cybervictimization and NSSI via negative emotions (β = −0.04, 95% CI [−0.09, −0.002])	Negative emotions were a partial mediator between cybervictimization and NSSI (β = 0.04, SE = 0.01, 95% CI [0.02, 0.06]).	Self-report: Friendship quality scale [106] and The Negative Emotion Scale [107]
CV and CA	Wiguna et al., 2021 [90], Indonesia	NSSI	6 months		Stress had a partial mediating effect on cyberbullying victimization and aggression in the relationship with adolescent NSSI (critical *t*-value: 5.27)	Self-report: adapted items from the Depression Anxiety Stress Scale-21 (DASS-21) [108]
CV	Yu et al., 2020 [26], China	NSSI	Past 6 months	-	School engagement mediated the relationship between cybervictimization and NSSI in subjects with high levels of attention-seeking (indirect effect = 0.018, SE = 0.008, 95% CI [0.005, 0.036])	Self-report: questionnaire [105]
CV	Zhao et al., 2022 [66], China	NSSI	Past 6 months	Dispositional mindfulness acted as a moderator for the direct effect of cyberbullying on NSSI (β = − 0.15, SE = 0.07, *p* < 0.05)	Emotion reactivity was a mediator between cyberbullying victimization and NSSI (β = 0.05, 95% CI = [0.031, 0.061])	Self-report: Child and Adolescent Mindfulness Measure (CAMM) [109] and the Emotion Reactivity Scale (ERS) [110]
CV	Zhu et al., 2021 [98], China	NSSI	12 months	Self-control moderated the association between anxiety symptoms and NSSI in cybervictims (β = −0.11, *p* < 0.05)	Anxiety symptoms mediated the relationship between cyberbullying and NSSI, but only in adolescents with low self-control (indirect effect = 0.04, *p* < 0.05, 95% CI [0.014, 0.083]).	Self-report: the Self-Control Scale [111] and the anxiety subscale in Depression Anxiety Stress Scales [112]

CV = cybervictimization; CA = cyberaggression. * refers to the period of time over which cyberbullying was investigated.

## Supplementary Materials

The following supporting information can be downloaded at: https://www.mdpi.com/article/10.3390/children11040410/s1, Figure S1: Identification of studies via databases and registers; Table S1: Search String; Table S2: Newcastle–Ottawa Scale rating.
