# Supplementary material for: Cyberbullying and Non-Suicidal Self-Injury (NSSI) in Adolescence: Exploring Moderators and Mediators through a Systematic Review
Source: Children (Basel). 2024 Mar 29;11(4):410. doi: 10.3390/children11040410 (PMC11049228; doi:10.3390/children11040410)
Supplement: Supplementary file 1 [file children-11-00410-s001.zip › Supplementary figure S1 - Identification of studies via databases and registers (PRISMA flowchart).pdf]

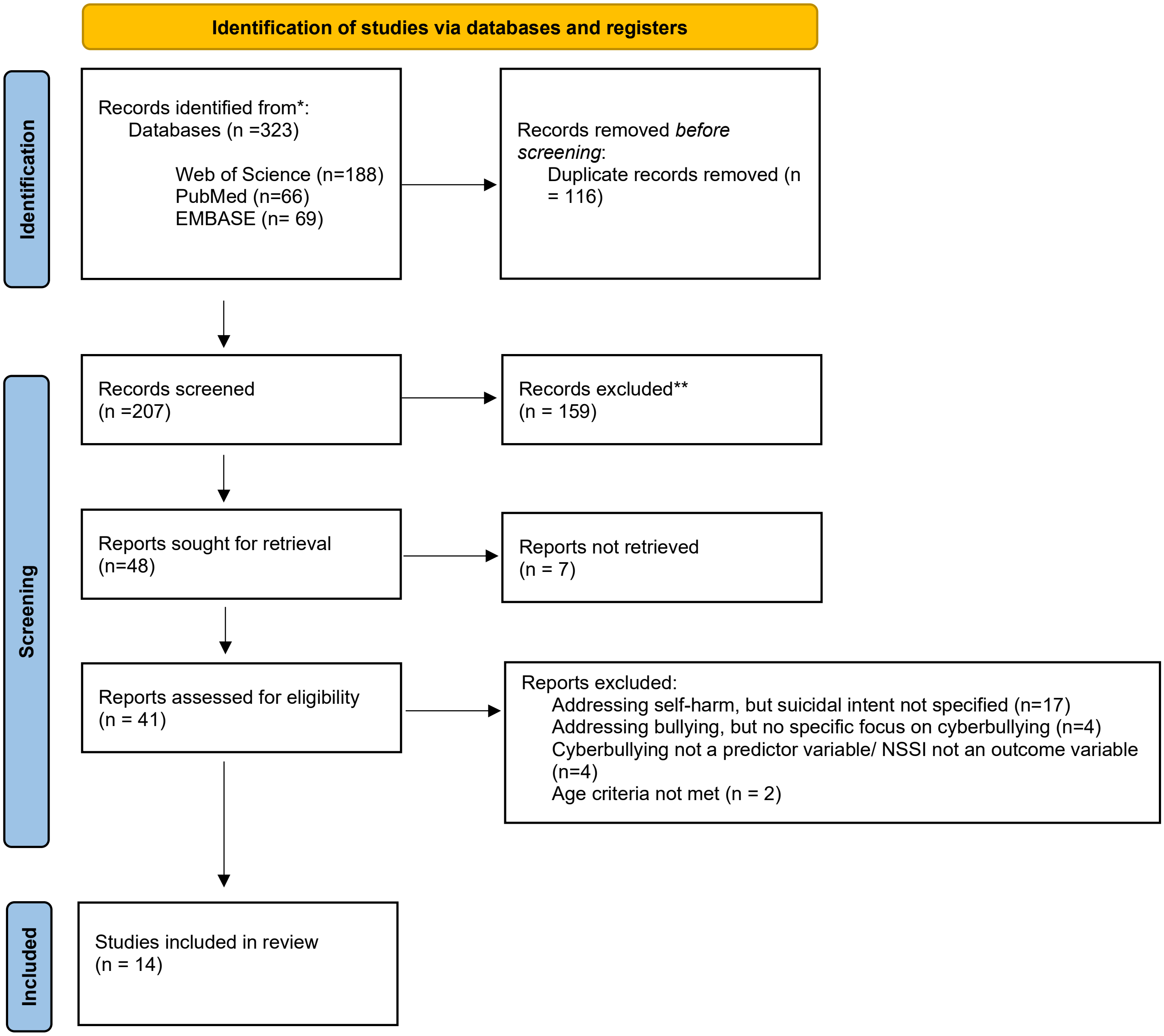

Figure S1. Identification of studies via databases and registers.

From: Page MJ, McKenzie JE, Bossuyt PM, Boutron I, Hoffmann TC, Mulrow CD, et al. The PRISMA 2020 statement: an updated guideline for reporting systematic reviews. BMJ 2021;372:n71. doi: 10.1136/bmj.n71
