# Supplementary material for: Cyberbullying and Non-Suicidal Self-Injury (NSSI) in Adolescence: Exploring Moderators and Mediators through a Systematic Review
Source: Children (Basel). 2024 Mar 29;11(4):410. doi: 10.3390/children11040410 (PMC11049228; doi:10.3390/children11040410)
Supplement: Supplementary file 1 [file children-11-00410-s001.zip › Supplementary Table S1 - SEARCH STRINGS.pdf]

Table S1. Search Strings.

WEB OF SCIENCE

(TS=(cyberbull) OR TS=(cyber-bull*) OR TS=(cybervictim*) OR TS=(cyber-victim*) OR TS=(cyber stalk*) OR TS=(cyber harass*) OR TS=(online bully*) OR TS=(internet bully*) OR TS=(electronic bully*) OR TS=(electronic harass*) OR TS=(online harass*) OR TS=(internet harass*) OR TS=(online stalk*) OR TS=(internet stalk*) OR TS=(electronic stalk*)) AND (TS=(self-harm*) OR TS=(NSSI) OR TS=(non suicidal self injury) OR TS=(self-injur*) OR TS=(self cut*) OR TS=(automutilat*)) AND (TS=(adolescen*) OR TS=(youth) OR TS=(child*) OR TS=(student*) OR TS=(school-aged))*

EMBASE

(cyberbullying:ti,ab,kw OR cybervictimization:ti,ab,kw OR 'online bully':ti,ab,kw OR cyberstalking:ti,ab,kw OR 'online harass*':ti,ab,kw OR 'online victim*':ti,ab,kw) AND (nssi:ti,ab,kw OR 'non-suicidal self-injury':ti,ab,kw OR 'self harm*':ti,ab,kw OR automutilation:ti,ab,kw) AND (youth:ti,ab,kw OR adolescent*:ti,ab,kw OR child*:ti,ab,kw OR 'school aged':ti,ab,kw OR 'student*':ti,ab,kw)*

other terms (e.g. cyber harass* electronic harass*) were indexed under terms or phrases from Emtree (e.g. online harass*), as suggested by the autocomplete function

PUBMED/MEDLINE

(((cyberbull[Title/Abstract]) OR (cyber bull*[Title/Abstract]) OR (cybervictim*[Title/Abstract]) OR (cyber victim*[Title/Abstract]) OR (cyber stalk*[Title/Abstract]) OR (cyber harass*[Title/Abstract]) OR (online bully*[Title/Abstract]) OR (internet bully*[Title/Abstract]) OR (electronic bully*[Title/Abstract]) OR (electronic harass*[Title/Abstract]) OR (online harass*[Title/Abstract]) OR (internet harass*[Title/Abstract]) OR (online stalk*[Title/Abstract]) OR (online victim*[Title/Abstract]) OR (internet stalk*[Title/Abstract]) OR (electronic stalk*[Title/Abstract]))) AND (((adolescen*[Title/Abstract]) OR (youth[Title/Abstract]) OR (child*[Title/Abstract]) OR (student*[Title/Abstract]) OR (school-aged[Title/Abstract]))) AND ((self harm*[Title/Abstract]) OR (non suicidal self injury[Title/Abstract]) OR (NSSI[Title/Abstract]) OR (self injur*[Title/Abstract]) OR (self cut*[Title/Abstract]) OR (self mutilat*[Title/Abstract]))*
