# Supplementary material for: Cyberbullying and Non-Suicidal Self-Injury (NSSI) in Adolescence: Exploring Moderators and Mediators through a Systematic Review
Source: Children (Basel). 2024 Mar 29;11(4):410. doi: 10.3390/children11040410 (PMC11049228; doi:10.3390/children11040410)
Supplement: Supplementary file 1 [file children-11-00410-s001.zip › Supplementary Table S2 -Newcastle-Ottawa Scale Rating.pdf]

<u>NOS Cross Sectional</u>

Selection:

1) Representativeness of the sample:

   a) Truly representative of the average in the target population (all subjects or random sampling) *

   b) Somewhat representative of the average in the target population (non-random sampling) *

   c) Selected group of users.

   d) No description of the sampling strategy.

2) Sample size:

   a) Justified and satisfactory. *

   b) Not justified.

3) Non-respondents:

   a) Comparability between respondents and non-respondents characteristics is established, and the response rate is satisfactory (>80%). *

   b) The response rate is unsatisfactory, or the comparability between respondents and nonrespondents is unsatisfactory.

   c) No description of the response rate or the characteristics of the responders and the non-responders.

4) Ascertainment of the exposure:

   a) Validated measurement tool (Cronbach's $\alpha > .05$). **

   b) Non-validated measurement tool, but the tool is available or described. *

   c) No description of the measurement tool.

Comparability:

1) The subjects in different outcome groups are comparable, based on the study design or analysis. Confounding factors are controlled.

   a) Study controls for gender/sex and age. *

   b) Study controls for any additional factor (e.g., depression, anxiety etc). **

   c) Not comparable on the basis of the design or analysis.

Outcome:

1) Assessment of the outcome:

   a) Independent blind assessment. **

   b) Record linkage. **

   c) Self report/interview. *

   d) No description.

2) Statistical test:

a) The statistical test used to analyse the data is clearly described and appropriate, and the measurement of the association is presented, including confidence intervals and the probability level (CI & P-value). *

b) The statistical test is not appropriate, not described, or incomplete.

<u>**NOS Cohort**</u>

Selection:

1) Representativeness of the exposed cohort:

a) Truly representative of the average in target population (all subjects or random sampling). *

b) Somewhat representative of the average in the target population (non-random sampling). *

c) Selected group of users.

d) No description.

2) Selection of the non-exposed cohort:

a) Drawn from the same community as the exposed cohort. *

b) Drawn from a different source.

c) No description.

3) Ascertainment of exposure:

a) Secure record (e.g., surgical records). *

b) Structured interview. *

c) Written self-report.

d) No description.

4) Demonstration that outcome of interest was not present at start of study:

a) Yes. *

b) No.

Comparability:

1) Comparability of cohorts on the basis of the design or analysis:

a) Study controls for gender/sex and age. *

b) Study controls for any additional factor (e.g., depression, anxiety etc). **

c) Not comparable on the basis of the design or analysis.

Outcome:

1) Assessment of outcome:

a) Independent blind assessment. *

b) Record linkage. *

c) Self-report/interview.

d) No description.

2) Was follow-up long enough for outcomes to occur:

a) Yes (e.g., 6 months or over). *

b) No.

3) Adequacy of follow up of cohorts:

a) Complete follow up - all subjects accounted for. *

b) Subjects lost to follow up but rate given (description given and <30%) *

c) Subjects lost to follow up (no description given and <70%).

d) No description at all.

Table S2. NOS for cross-sectional studies.

**Author (year)**	**Selection**				**Comparability**	**Outcome**		**Total Score**
	**Q1**	**Q2**	**Q3**	**Q4**	**Q1**	**Q1**	**Q2**	
Azami and Taremian 2023)	★		★	★★		★	★	6
Drubina et al. (2023)			★	★	★	★	★	5
Islam et al. (2020)	★			★★	★	★	★	6
Islam et al. (2021)	★			★		★	★	4
Lanzillo et al. (2023)				★★	★	★	★	5
Liu et al. (2023)	★		★	★★	★★	★	★	8
Peng et al. (2019)	★			★★	★★	★	★	7
Wang et al. (2021)			★	★★	★	★	★	6
Wiguna et al. (2021)				★★		★	★	4
Wright and Wachs (2020)		★	★	★★	★★	★	★	8
Yu et al. (2020)	★		★	★★	★★	★	★	8
Zhao et al. (2021)		★	★	★★	★★	★	★	8

NOS for cohort studies

**Author**	**Selection**				**Comparability**	**Outcome**			**Total Score**
	**Q1**	**Q2**	**Q3**	**Q4**		**Q1**	**Q2**	**Q3**	
Lin et al. (2023)		★	★		★★		★	★	6
Zhu et al. (2021)	★	★	★		★★		★	★	7
